# Leaf spectroscopy of resistance to Ceratocystis wilt of ‘Ōhi’a

**DOI:** 10.1371/journal.pone.0287144

**Published:** 2023-06-23

**Authors:** Megan M. Seeley, Roberta E. Martin, Christian Giardina, Blaine Luiz, Kainana Francisco, Zachary Cook, Marc A. Hughes, Gregory P. Asner

**Affiliations:** 1 Center for Global Discovery and Conservation Science, Arizona State University, Hilo, Hawaiʻi, United States of America; 2 School of Geographical Sciences and Urban Planning, Arizona State University, Tempe, Arizona, United States of America; 3 Institute of Pacific Islands Forestry, Pacific Southwest Research Station, USDA Forest Service, Hilo, Hawaiʻi, United States of America; 4 Akaka Foundation for Tropical Forests, Hilo, Hawaiʻi, United States of America; Universita degli Studi di Pisa, ITALY

## Abstract

Plant pathogens are increasingly compromising forest health, with impacts to the ecological, economic, and cultural goods and services these global forests provide. One response to these threats is the identification of disease resistance in host trees, which with conventional methods can take years or even decades to achieve. Remote sensing methods have accelerated host resistance identification in agricultural crops and for a select few forest tree species, but applications are rare. Ceratocystis wilt of ʻōhiʻa, caused by the fungal pathogen *Ceratocystis lukuohia* has been killing large numbers of the native Hawaiian tree, *Metrosideros polymorpha* or ʻŌhiʻa, Hawaii’s most common native tree and a biocultural keystone species. Here, we assessed whether resistance to *C*. *lukuohia* is detectable in leaf-level reflectance spectra (400–2500 nm) and used chemometric conversion equations to understand changes in leaf chemical traits of the plants as indicators of wilt symptom progression. We collected leaf reflectance data prior to artificially inoculating 2–3-year-old *M*. *polymorpha* clones with C. *lukuohia*. Plants were rated 3x a week for foliar wilt symptom development and leaf spectra data collected at 2 to 4-day intervals for 120 days following inoculation. We applied principal component analysis (PCA) to the pre-inoculation spectra, with plants grouped according to site of origin and subtaxon, and two-way analysis of variance to assess whether each principal component separated individuals based on their disease severity ratings. We identified seven leaf traits that changed in susceptible plants following inoculation (tannins, chlorophyll a+b, NSC, total C, leaf water, phenols, and cellulose) and leaf chemistries that differed between resistant and early-stage susceptible plants, most notably chlorophyll a+b and cellulose. Further, disease resistance was found to be detectable in the reflectance data, indicating that remote sensing work could expedite Ceratocystis wilt of ʻōhiʻa resistance screenings.

## Introduction

Native dominated forest ecosystems around the world are being impacted, and in some cases decimated by diseases caused by non-native pests and pathogens [1–3]. Instances of virulent and problematic forest pathogens have been rising and are predicted to continue increasing due to climate change [4, 5], globalization [6], and reduced resilience resulting from genetic diversity loss [7, 8]. Ecological, economic, and social consequences of these pathogens necessitate resistance screening, breeding, and restoration programs to mitigate the effect of these diseases [9–12]. Successful disease resistance programs have established germplasm that exhibit higher survival rates than non-resistant material in the ecologically and economically important *Pinus monticola* (western white pine) to the disease white pine blister rust (*Cronartium ribicola*) [12] and *Ulmus americana* (American elm) cultivars to Dutch elm disease (*Ophiostoma novo-ulmi*) [13]. As these programs are usually long-term, labor-intensive investments [14], remote sensing has emerged as a means of greatly expanding pre-screening efforts by discriminating between resistant and susceptible phenotypes in trees [15–17] and disease monitoring [18].

Remote sensing technologies are an asset to conservation in the face of forest pathogens as they can be used to better understand patterns of disease progression and determining resistant individuals [15, 19–21]. While remote sensing has long been used to aid in forest monitoring and management [22, 23], advances in spectroscopy, or hyperspectral remote sensing, have allowed us to classify species [24–26], monitor biodiversity [27], and distinguish between subspecies [28, 29]. The connection between plant traits, phylogenetics, and spectroscopy is fundamental to spectranomics, which allows us to understand ecological processes at large scales [30, 31].

Spectroscopy data can be used to describe plant chemical (e.g. chlorophyll a and b, lignin, phenols, tannins, nonstructural carbohydrates, total carbon, total nitrogen) and structural traits (e.g. leaf mass per area) at high spectral resolution (1–10 nm). Some methods use the entire visible to shortwave infrared (VSWIR) spectra (350–2500 nm) while others capture only the visible to near infrared (VNIR; 350–1050 nm) spectra. Not only have traits captured in spectroscopic data been used to understand the genetic structure of plants and their evolutionary history [30, 32, 33], but reflectance spectra have been shown to distinguish individuals based on their resistance to diseases [16, 20, 34–37]. For example, spectroscopy is a promising method for both determining putatively disease resistant germplasm and nondestructively monitoring changes in leaf traits. Spectroscopy of *Ulmus* twigs was used to identify individuals resistant to Dutch elm disease [17], and spectral measurements of *Pinus taeda* (loblolly pine) during resistance screenings against fusiform rust likewise allowed resistant and susceptible individuals to be distinguished [35].

Ceratocystis wilt of ʻōhiʻa, caused by the non-native fungus *Ceratocystis lukuohia*, poses an unprecedented conservation concern in Hawaiʻi, as the Hawaiian endemic tree, ʻōhiʻa (*Metrosideros polymorpha*) [38, 39] is an eclogical and cultural keystone species that accounts for approximately fifty percent of the biomass in forested areas across the Hawaiian Islands [40] and provides habitat to many endemic plants and animals [41]. Further, *M*. *polymorpha* is often a forest dominant making up 80% of native forest basal area [11], and so sustains vital ecosystem services as it contributes more to groundwater recharge than non-native forests [42]. In addition to its ecological importance, *M*. *polymorpha* is culturally significant to Hawaiian people [43, 44], is Hawaii’s state endemic tree, and has become a cherished presence in residential settings. For these reasons, many Hawaiian organizations, in collaboration with academic and government organizations, are dedicated to protecting this species.

In the ten years since its discovery, the Ceratocystis wilt of ʻōhiʻa appears to spread easily and rapidly, with widespread mortality of *M*. *polymorpha* trees across Hawaiʻi Island and occurences on Kauai, Oʻahu, and Maui [45, 46]. Of equal concern is that *C*. *lukuohia* has caused up to 90% mortality in many *M*. *polymorpha* stands [47]. *C*. *lukuohia* is a wilt pathogen that affects the vascular system of its hosts, resulting in canopy wilt, rapid browning and eventual death [39]. Ecological consequences of *C*. *lukuohia*-caused mortality include declines in endemic avifauna and plants [48, 49], and research in this field is rapidly developing to understand and combat this new threat [50–52].

Hawaiʻi is home to a very large number of non-native invasive plant species [53–55], and so the loss of *M*. *polymorpha* in forests can result in a proliferation of invasive plants, which accelerates the conversion of native dominated forests to non-native forests, with detrmental impacts to stand and watershed hydrology, and to native fauna [56–59]. The need for immediate action is hampered by the long timelines defining resistance programs, but remote sensing applications have the potential to accelerate disease resistance screening methods. Current research efforts are focused on monitoring disease spread and developing management strategies to combat *Ceratocystis*-induced *M*. *polymorpha* mortality [52, 60, 61] as well as resistance screening [47, 51]. In 2018, the USDA Forest Service (USFS) in partnership with the USDA Agricultural Research Service and Akaka Foundation for Tropical Forests established the ʻŌhiʻa Disease Resistance Program (ʻŌDRP; www.akakaforests.org/projects/ohia-disease-resistance-program) in order to identify, propagate, and outplant individuals resistant to *C*. *lukuohia* with the goal of restoring regions affected by this pathogen [47, 51]. Identification of potentially resistant material for propagation is entirely ground-based and not based on genetically or environmentally determined quantatitive measures. Rather, collections have been focused on random sampling of live trees in intact forests or from live individuals within heavily disease-impacted stands. As a result, the identification of resistant individuals has been time and labor intensive.

Streamlining the identification of disease resistant individuals would lead to the more rapid identidication of candidate trees for resistance screening, where remote sensing-based pre-screening would narrow down the potential set of candidate trees for follow up sampling and inoculation-based screening. To address the limitations of existing resistance sampling methodologies and assess the potential of remote sensing based pre-screeing of *M*. *polymorpha* trees, we tested the applicability of spectroscopic data in screening for Ceratocystis wilt of ʻōhiʻa resistance. Relying on ʻŌDRP inoculation screening trials, our objective was to monitor leaf spectra of *M*. *polymorpha* clones before and after artificial inoculation to determine if there is a spectral signature that can discriminate between clones that are classified as susceptible or resistant based on external disease symptoms. Additionally, we investigated whether chemometric conversion equations could be useful in understanding changes in leaf chemical traits of the plants as indicators of wilt symptom progression.

## Materials and methods

### Field collections

**Fig 1:**
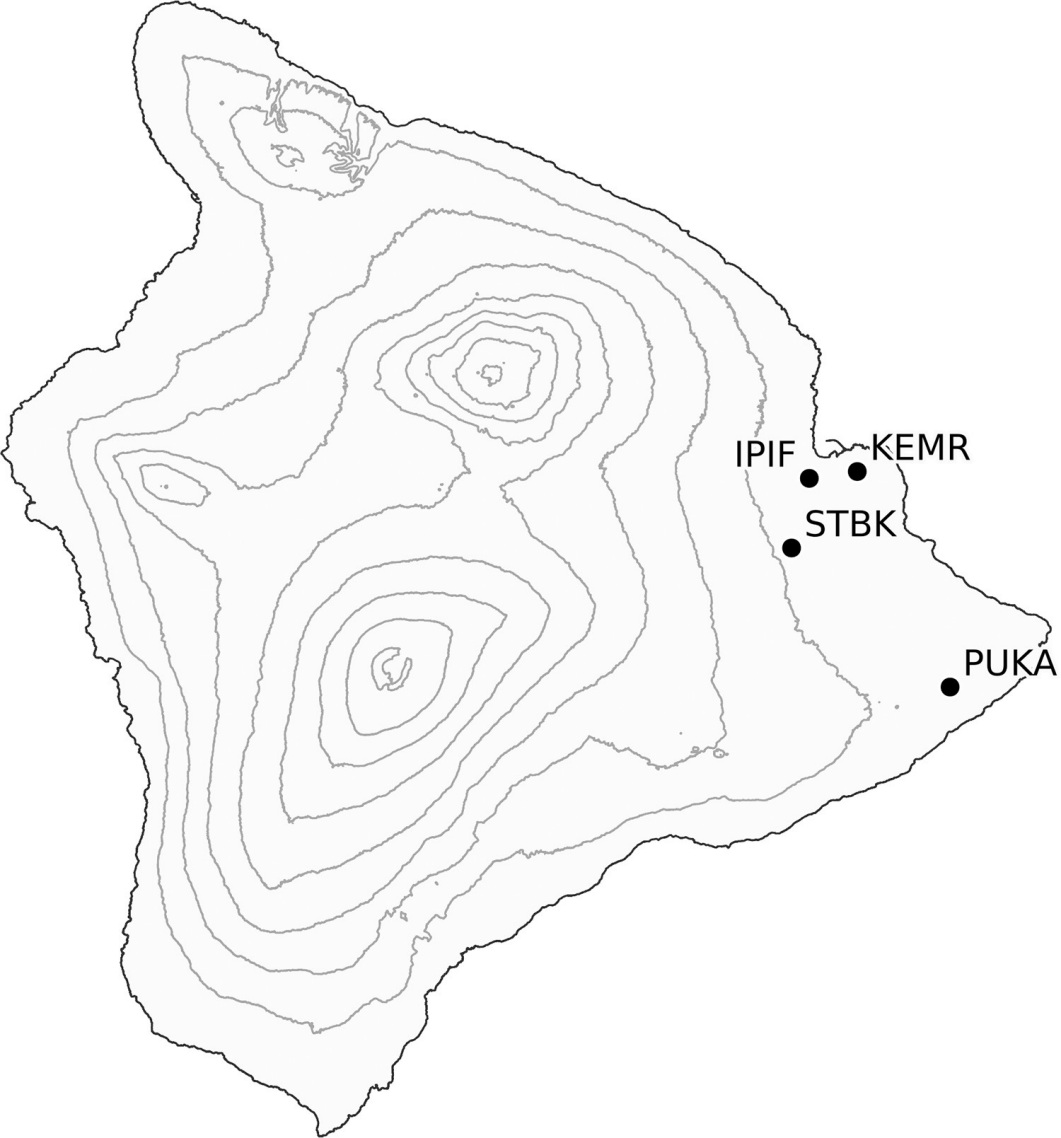
Locations of the four study sites across the windward side of Hawaiʻi Island. Sites where putatively *Ceratocystis lukuohia*-resistant (asymptomatic) *Metrosideros polymorpha* trees were sampled for vegetative propagation to generate clonal ramets. Topographic lines represent 500-meter elevational increments derived from the Shuttle Radar Topography Mission Digital Elevation Model. The four sites represented in this study are: Institute of Pacific Islands Forestry (IPIF), Keaukaha Military Reserve (KEMR), Stainback Highway (STBK), and Puʻu Kaliu (PUKA).

Vegetative cuttings were collected from 53 putatively disease resistant *M*. *polymorpha* trees (mother trees) located across four sites on the windward side of Hawaiʻi Island in 2019 and 2020 (Fig 1; Table 1; S1 Table). The sites included the USDA Forest Service’s Institute of Pacific Islands Forestry (IPIF; -155.0955, 19.6977), Keaukaha Military Reserve (KEMR; -155.0376, 19.7069), and Stainback Highway (STBK; -155.1180, 19.6133) all in the district of Hilo, and Puʻu Kaliu (PUKA; -154.9269, 19.4489) in the district of Puna. Permits for access, collecting and research were obtained from the State of Hawaiʻi, Department of Land and Natural Resources for the STBK site, the PUKA site by Kamehameha Schools^®^, and the KEMR site by the Hawaiʻi Army National Guard- Environmental Office. No permits were needed to access and collect plant material from the IPIF site. Trees were selected from forest stands exhibiting either high or low levels of *C*. *lukuohia*-induced mortality at each of the four sites. On Hawaiʻi Island, *M*. *polymorpha* has four distinct genetic varieties [62–65], two of which were present at the collection sites. Cuttings from *M*. *polymorpha* var. *glaberrima* and *incana* were collected as well as hybrids of those two varieties, hereafter referred to as *incana*, *glaberrima*, and the hybrid (*incana x glabberima*), respectively. Variety was determined based on leaf morphology, with degree of pubescence being the most distinguishing factor [65–67]. *Glaberrima* leaves are glabrous while *incana* leaves are pubescent; hybrids are pubescent, but unlike *incana*, leaf hairs are easily removed via rubbing [65–67]. After collection, cuttings were vegetatively propagated in a greenhouse under mist at the IPIF in Hilo, Hawaiʻi. We succeeded in raising 188 clonal ramets for the inoculation trails in 2021. Each tree from which cuttings were collected are hereafter referred to as “mother trees” with mother trees being represented by 1 to14 clonal ramets in the inoculation trials. Clones were maintained in 10.2 cm x 24.4 cm band pots (Anderson Pots, Portland, Oregon, USA) in a greenhouse, with plants receiving drip irrigation twice a day.

**Table 1:** *Metrosideros polymorpha* mother trees used for vegetative propagation and number of generated clonal ramets used in *Ceratocystis lukuohia* inoculation trials. Trees are denoted by species varieties *incana*, *glaberrima* or their hybrid and site location. Counts are listed for all trees that had their leaf spectra sampled pre-inoculation (Pre-Inoc.) as well as those regularly sampled post-inoculation (Post-Inoc.). The four sites from Hawaiʻi Island represented in this study are: Institute of Pacific Islands Forestry (IPIF), Keaukaha Military Reserve (KEMR), Stainback Highway (STBK), and Puʻu Kaliu (PUKA). See S1 Table for site descriptions.

		No. Mother Trees		No. Clonal Ramets	
Variety	Site	Pre-Inoc.	Post Inoc.	Pre-Inoc.	Post Inoc.
*glaberrima*	IPIF	2	2	21	11
	PUKA	16	7	51	32
	STBK	18	14	29	16
hybrid	IPIF	2	2	22	11
	KEMR	5	3	17	12
	PUKA	2	2	7	7
*incana*	IPIF	1	1	9	6
	KEMR	3	2	5	3
	PUKA	3	2	22	13
	STBK	1	1	5	1

### Inoculations

In October 2021, all clones were transported to a nearby greenhouse at the University of Hawaiʻi at Mānoa’s Komohana Research and Extension Center in Hilo, HI and inoculated with either *C*. *lukuohia* or sterile water as a negative control. To make inoculum, 7-day-old *C*. *lukuohia* cultures (fungal isolate PL1959) grown on 10% V8 media agar plates, were flooded with sterile water, the surface scraped with a sterile glass rod to dislodge fungal conidia (spores) and collected in a beaker. This spore suspension was quantified to 1.0 x 10^6^ spores/ml with a hemacytometer. Before inoculation, 20 μl of the spore suspension (equivalent to 20,000 spores) was removed by pipette and dropped onto sterile filter paper discs (Whatman, GE Healthcare UK Limited, Buckinghamshire, UK) cut to 6mm in diameter. To inoculate, clones were wounded with a sterile scalpel on the main stem 2 cm above soil level at a downward angle to expose fresh sapwood. The *C*. *lukuohia* saturated filter disc was then placed in the wound, the stem-flaps closed and the wound site wrapped in laboratory wax film (Parafilm, Bemis Company, Inc, Neenah, Wisconsin) [68]. External symptom progression of inoculated clones was evaluated approximately two to three times per week, according to a 0–5 disease severity scale where 0 = fully asymptomatic, 1 = 1 to 25% foliar crown wilt, 2 = 26 to 50%, 3 = 51 to 75%, 4 = 76 to 99% and 5 = plants being completely dead (100% wilted). Individual clones that remained asymptomatic throughout the 127-day trial were hereby labeled as “*Resistant*” (final rating of 0) while those that showed any wilt symptoms were labeled as “*Susceptible*” (final rating of 1 to 4). The resistance rate was calculated based on the percentage of clones per mother tree that were asymptomatic after inoculation. The effect of site and genotype on resistance rates, independent of leaf spectra or chemistry, were assessed using a two-way analysis of variation (ANOVA; S2 Table).

### Leaf reflectance sampling

Leaf reflectance spectral measurements were taken pre- and post-inoculation using an Analytical Spectra Devices plant probe, leaf clip, and FieldSpec spectroradiometer (Analytical Spectra Devices Inc., Boulder, CO, USA), which allowed us to make measurements on live vegetation, and field spectrometer at 1 nm intervals from 350 to 2500 nm (S1 Fig). One set of measurements were taken 1 to 2 days prior to inoculation with *C*. *lukuohia*. The post-inoculation measurements for leaf spectra were taken every 2 to 4 days for 106 days then reduced to once per week until the study ended at 127 days. Three leaves were selected from each plant, wiped to remove sooty mold and dust using distilled water, and patted dry prior to taking spectral measurements. Throughout the inoculation trial, the same three leaves were used for measurements, and so great care was taken to not damage these leaves. In instances where leaves were damaged or when leaves died, we selected replacements that were most like the lost leaf. As estimated chemistry profile of the leaves from the same plant were less variable than those between plants (one-way ANOVA applied to leaf-level pre-inoculation data), indicating that replacing fallen leaves likely had no or minimal effect on the results. To maintain consistency regarding time of day, spectra were collected between 8am and 2pm. A white reference was used to calibrate spectra every 20 to 30 minutes. Parabolic corrections were applied *post-hoc* to minimize differences in spectral measurements resulting from temperature sensitivities of one of the sensors. The differential temperature sensitivities of sensors in the spectrometer causes a jump in the spectra at 1000 nm, which were corrected according to Hueni & Bialek [69]. Spectra were trimmed to 400–2450 nm to minimize noise. Brightness normalization was applied to minimize between sample variation in illumination.

Pre-inoculation sampling included all 188 clonal ramets, but we tracked 112 of these plants post-inoculation due to logistical constraints. Approximately half of the *M*. *polymorpha* genotypes we measured were of the variety *glaberrima*. Of the four sites, PUKA, followed by STBK, had the most mother trees sampled to make clonal ramets (Table 1). Clones selected for spectral sampling post-inoculation represented all four sites as well as both genetic varieties and their hybrid. Five individual ramets per mother tree were sampled to capture the variation within each mother tree. For mother trees with fewer than five ramets, one replicate was sampled to increase our geographic range; these trees were most commonly from the STBK site (Table 1). Twelve of the 21 plants inoculated with sterile water infused discs as negative controls were sampled post-inoculation.

### Analysis

To determine if spectroscopy could be used to distinguish *C*. *lukuohia Resistant* (n = 87) individuals from *Susceptible* (n = 101) individuals, we examined the separability of leaf spectra from these two groups using pre-inoculation reflectance data using a modified version of the methods used by Cavender-Bares [29]. As variations in reflectance spectra of *M*. *polymorpha* grown in common gardens result from both environmental filtering and genotype [70], we grouped individuals first by mother tree collection site and then by species variety. Sample sizes were insufficient to separate data by both factors simultaneously. Reflectance data were converted using principal component analysis (PCA) to obtain a lower dimensional dataset that expresses information relevant to biological diversity [71] using the *pca* function from the *scikit learn* python v. 3.6.9 [72]. PCA is commonly used in spectroscopy applications to reduce dimensionality, and the resulting PC have been shown to correspond to ecologically-relevant metrics such as diversity [71, 73] and canopy structure [74]. We then assessed whether any of the principal components (PC) separated clones between *Resistant* and *Susceptible* mother trees using a two-way ANOVA using the *statsmodels* python package [75]. The number of PC was limited to the sample size of each group. The two-way ANOVA assessed the influence of either collection site or genotype/variety on *C*. *lukuohia* symptom expression, and the interaction of ROD susceptibility and either site or genotype. Two-way ANOVAs were applied to data representing each variety and site, respectively. We additionally assessed the separability of plant reflectance data by site and genotype using PCA, one-way ANOVA, Tukey HSD pairwise comparison to significant PC according to the ANOVA (S3, S4 Tables). Statistical significance was defined as *p* < 0.05.

Next, we assessed ROD symptom progression via leaf chemical constituent indices derived from reflectance spectra. Eight leaf chemical constituent indices, hereafter referred to as leaf chemistries, were estimated from the reflectance data using spectro-chemometric equations developed by Asner et al. for *M*. *polymorpha* [50]. These conversion equations use partial least squares regression (PLSR) to estimate percent leaf water, nonstructural carbohydrates (NSC) such as sugars and starches, total nitrogen (N), total carbon (C), and chlorophylls a and b (summed here and referred to as chlorophyll a+b). These equations additionally estimated structural (lignin and cellulose) and secondary (phenols and tannins) compounds. This method has been used to develop universal broadleaf spectro-chemical equations [30, 76]. We note that the chemometric equations developed by Asner et al. [50] used an integrating sphere while we used a plant probe and leaf clip, which may result in minor offsets in estimated chemistry values. As the chemistries were used in a comparative analysis, the slight differences between our values and those of Asner et al. [50] did not affect the interpretation of the results. Leaf chemistries were binned according to the disease symptom scale ratings of the plant at the time of measurement. For this, we pooled all post-inoculation sampling points. As the rate of ROD progression differed for each inoculated clone, this method allowed us to control for differences in time to symptom onset. We used a one-way ANOVA to determine whether leaf chemical trait values differed across symptom progression levels.

Lastly, we sought to detect early leaf chemistry indicators of ROD susceptibility. To do this, we compared the leaf chemistry of *Susceptible* individuals at disease progression levels 0 and 1 (asymptomatic and early-stage symptom onset) to *Resistant* individuals across time. We first normalized data at all time points using the negative control plants. Then we plotted the mean deviation from pre-inoculation trait values for each group over time. To detect significant differences between plants that succumbed to ROD and those that were classified as *Resistant*, we performed a t-test using the *scipy* python package at each sampling time point [72].

## Results

**Fig 2:**
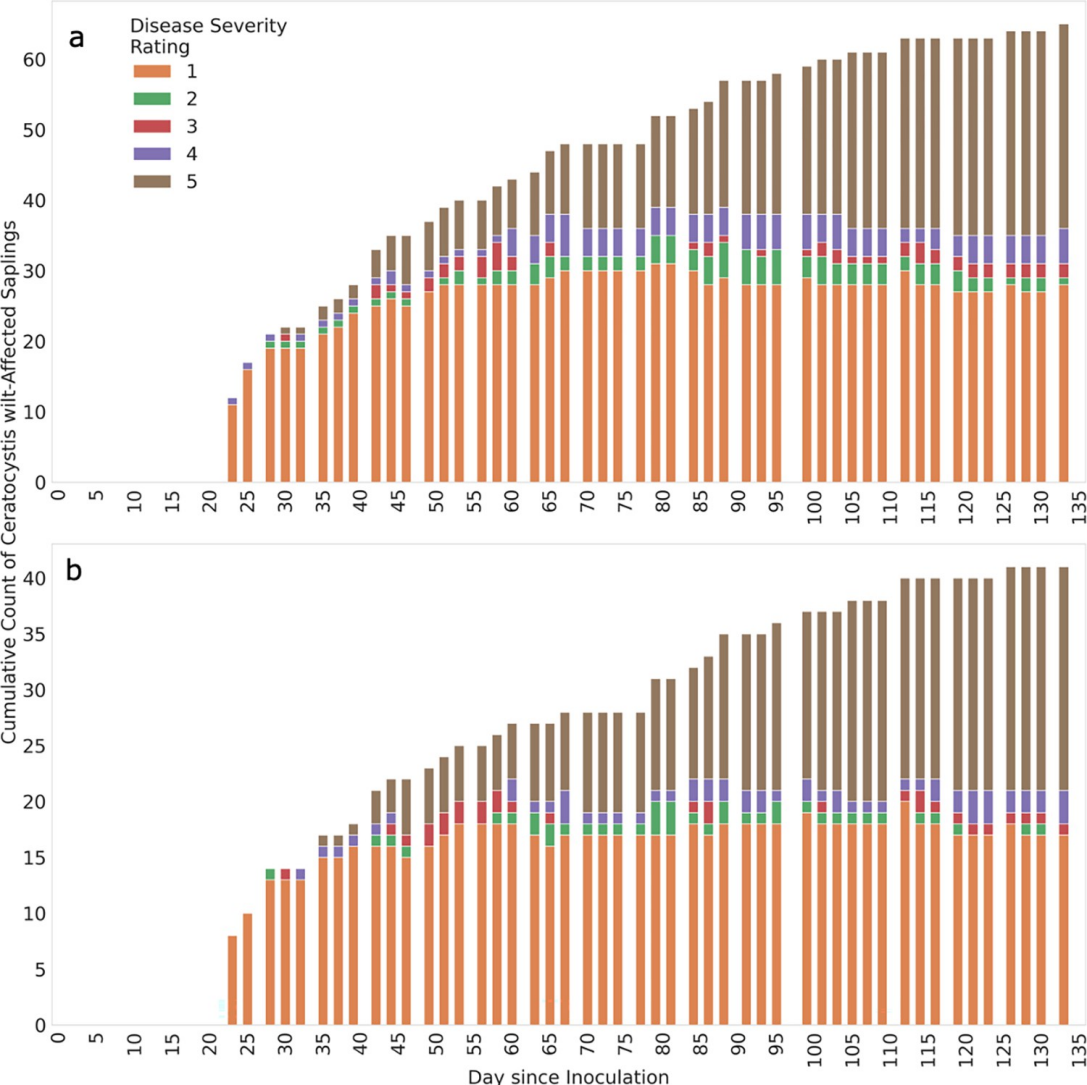
Cumulative counts by day of symptomatic (wilted) *Metrosideros polymorpha* clones after artificial inoculation by *Ceratocystis lukuohia*. (A) Counts for all plants sampled pre-inoculation and (B) counts for plants sampled post-inoculation. Stacked histograms are colored based on disease progression level. Mean disease severity (wilt) ratings are as follows: where 0 = fully asymptomatic, 1 = 1 to 25% foliar crown wilt, 2 = 26 to 50%, 3 = 51 to 75%, 4 = 76 to 99% and 5 = plants being completely dead (100% wilted).

**Fig 3:**
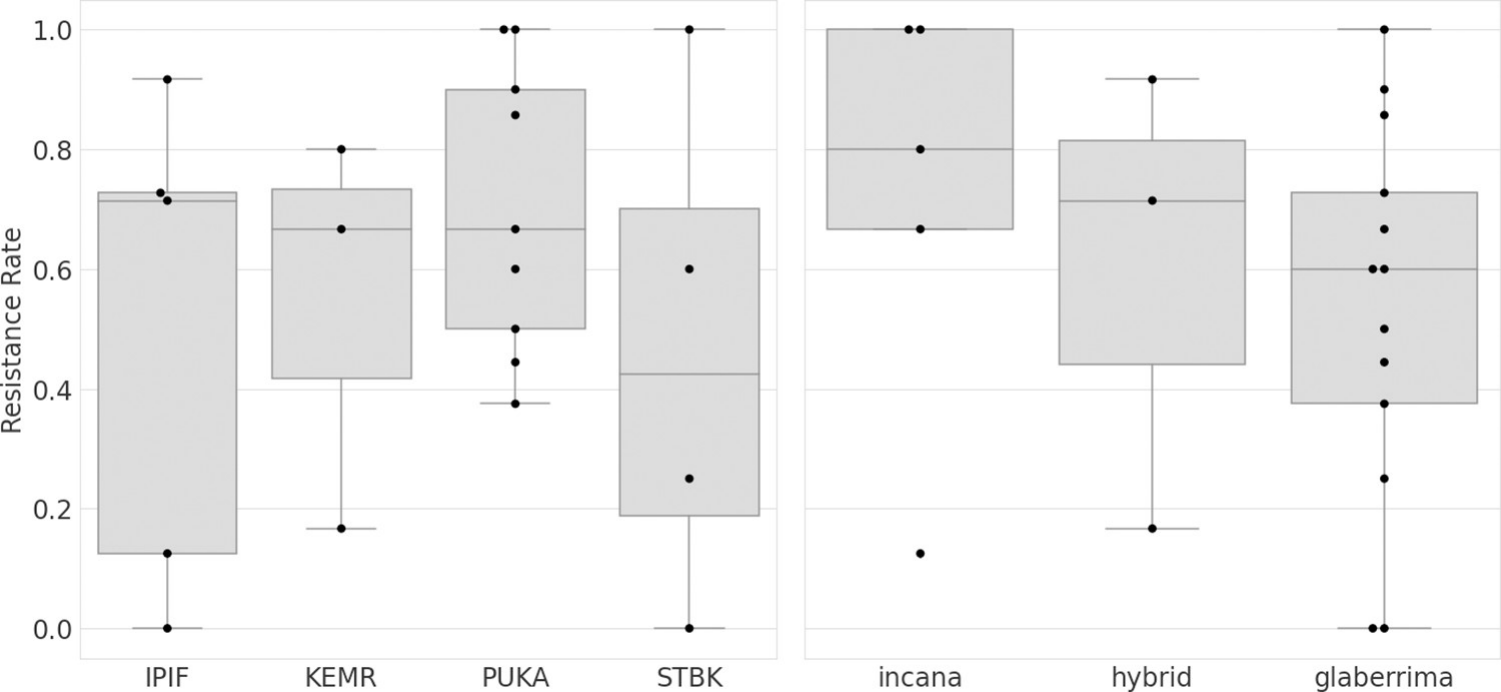
Boxplot showing proportion of asymptomatic *Metrosideros polymorpha* clones after artificial inoculation with *Ceratocystis lukuohia*. “Resistance Rate” is denoted as (A) the range of proportion of asymptomatic (disease rating of 0) clones per mother tree collection site or (B) range of proportions of asymptomatic clones per plant variety. Analysis of variation (ANOVA) indicated that differences in resistance rate are not significant. Site locations and their abbreviations on Hawaiʻi Island are denoted as: Institute of Pacific Islands Forestry (IPIF), Keaukaha Military Reserve (KEMR), Stainback Highway (STBK), and Puʻu Kaliu (PUKA) in the district of Puna.

A total of 65 (35%) of the initial 188 clones died (severity rating of 5) due to *C*. *lukuohia* inoculation by the end of the 127-day trial. Thirty-six individuals showed wilt symptoms (rating 1 to 4) but had yet to progress to disease progression stage 5 (Fig 2A), with the remaining 87 clones showing no sign of disease (severity rating of 0). Of the 112 individuals sampled for leaf reflectance throughout the inoculation trial, 41 were Ceratocystis wilt-symptomatic at a variety of disease progression levels, with 20 having died during the trial (Fig 2B). The influence of site, variety, or their combination on Ceratocystis wilt resistance were not significant (*P* = 0.059 to *P* = 0.147; Fig 3; S2 Table). While not a significant predictor of resistance, site and genotype did affect foliar spectra. Principal components (PC) of leaf reflectance spectra separated all variety (S3 Table) and site (S4 Table) pairings.

### Spectroscopy of disease resistance

Putative Ceratocystis wilt resistance was detectable in leaf spectra using PCA and ANOVA, but sample sizes of clones were insufficient to assess the detectability of a resistance signal in the spectra within one genotype from one collection site. We therefore evaluated individuals grouped first by collection site then variety using two-way ANOVA. While both site and plant variety had significant effects on leaf spectra, we present only results where disease resistance data or the interaction of either site x resistance or genotype x resistance were significant. When grouped by variety, spectra of *incana* resistant and susceptible individuals were separable in PC 1, while the interaction of resistance x site was significant in PC 12 (Table 2). PC 1 additionally separated individuals by site. The hybrid had only one significant PC (25) based on resistance (Table 2). The variety *glaberrima* had a larger sample size (Table 1) as well as more significant PCs than the other varieties. PCs 3 and 5 significantly separated *Resistant* and *Susceptible* clones; site was also significant at these PCs (Table 2). The interaction of resistance x site was significant at PC 12 and 25 (Table 2).

**Table 2:** Results of two-way analysis of variance (ANOVA) assessing whether principal components (PC) of leaf reflectance data differentiate *Metrosideros polymorpha* that are *Resistant* and *Susceptible* to *Ceratocystis lukuohia* by artificial inoculation. Data were alternately grouped by species variety (left) and site of mother tree origin (right), and groupings are indicated by bolded headings. Only PCs that were significant for either resistance or the interaction between resistance and site or variety are shown. Site locations and their abbreviations on Hawaiʻi Island are denoted as: Institute of Pacific Islands Forestry (IPIF), Keaukaha Military Reserve (KEMR) and Puʻu Kaliu (PUKA) in the district of Puna.

	F	p-value		F	p-value
***Incana***			**IPIF**		
PC 1			PC 7		
Resistance	9.416	0.005	Resistance	4.420	0.042
Site	13.437	0.000	Variety	6.000	0.005
Resistance x Site	0.286	0.754	Resistance x Variety	0.423	0.658
PC 12			PC 21		
Resistance	0.143	0.709	Resistance	5.758	0.021
Site	0.143	0.868	Variety	3.083	0.057
Resistance x Site	3.576	0.042	Resistance x Variety	1.972	0.153
**Hybrid**			PC 23		
PC 25			Resistance	2.246	0.142
Resistance	9.398	0.004	Variety	1.940	0.157
Site	0.502	0.609	Resistance x Variety	3.702	0.034
Resistance x Site	1.791	0.182	**PUKA**		
***Glaberrima***			PC 1		
PC 3			Resistance	4.147	0.046
Resistance	7.368	0.008	Variety	2.449	0.094
Site	3.580	0.032	Resistance x Variety	1.673	0.195
Resistance x Site	1.186	0.311	PC 5		
PC 5			Resistance	0.179	0.673
Resistance	4.957	0.029	Variety	1.438	0.245
Site	7.164	0.001	Resistance x Variety	3.323	0.042
Resistance x Site	1.119	0.331	PC 23		
PC 12			Resistance	4.859	0.031
Resistance	3.163	0.079	Variety	0.081	0.923
Site	7.564	0.001	Resistance x Variety	1.009	0.370
Resistance x Site	4.138	0.019	**KEMR**		
PC 25			PC 4		
Resistance	0.014	0.907	Resistance	0.001	0.975
Site	0.194	0.824	Variety	1.838	0.194
Resistance x Site	4.704	0.012	Resistance x Variety	5.283	0.035
			PC 11		
			Resistance	16.514	0.001
			Variety	0.919	0.352
			Resistance x Variety	0.258	0.619

Reflectance spectra grouped by site separated clonal ohia genotypes based on ROD survivorship for three of the four sites. We found no significant patterns in reflectance spectra based on resistance at the STBK site (results not shown). IPIF had two PCs, 7 and 21, that separated the data based on resistance, and one (PC 23) that separated the data by the interaction of site x genotype (Table 2). PC 7 additionally distinguished spectra based on variety. PUKA had two PCs that distinguished resistant from susceptible individuals (PC 1 and 23), and the interaction between resistance x genotype was significant in PC 5 (Table 2). Lastly, resistance was significant in PC 11 for KEMR while the interaction of resistance x genotype was significant in PC 4.

### Rod symptom progression

**Fig 4:**
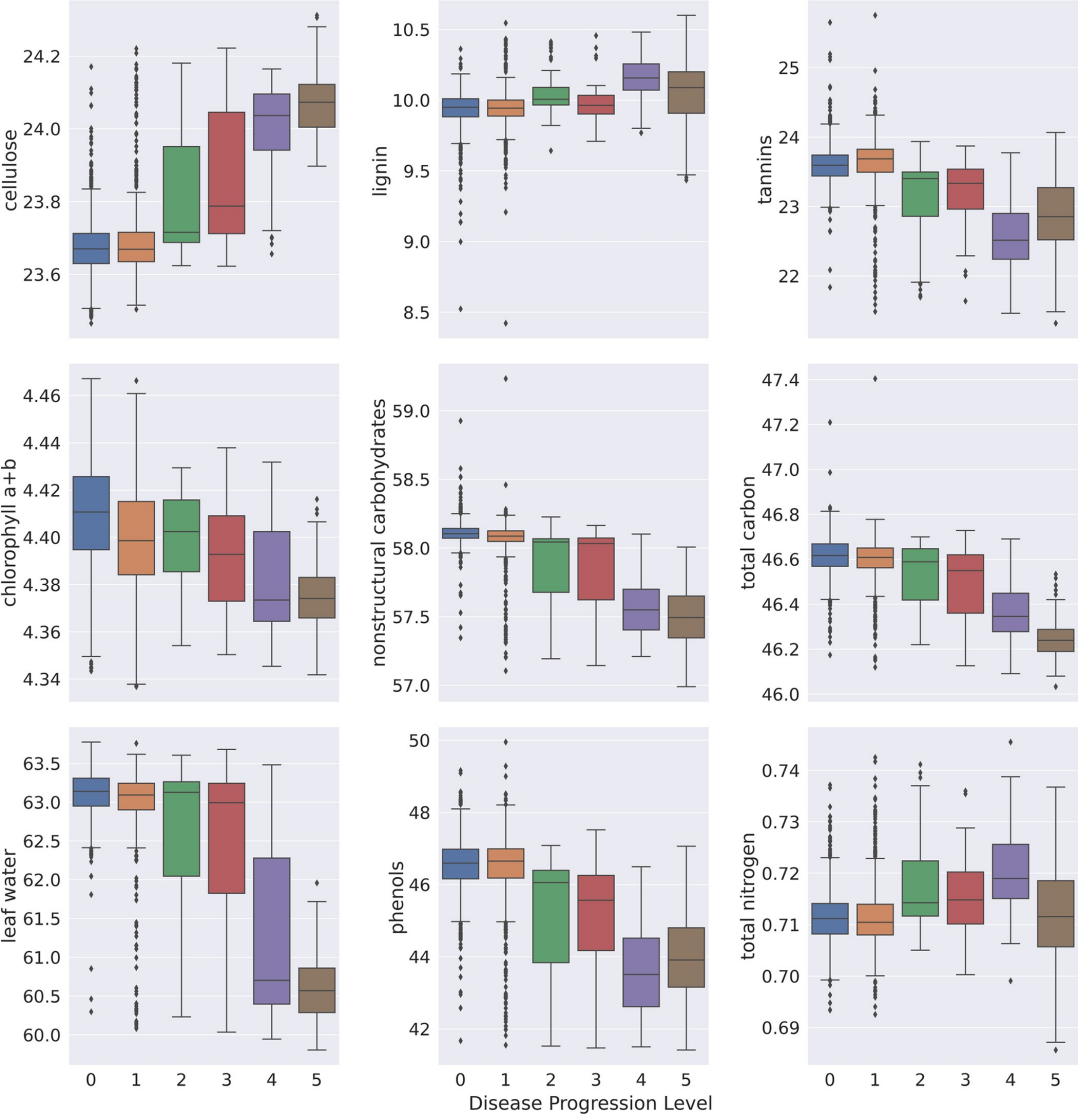
Boxplots of the nine *Metrosideros polymorpha* leaf traits derived from reflectance spectra according to Asner et al. [50]. Trait values are displayed by disease severity ratings after artificial inoculation with *Ceratocystis lukuohia*. Differences across disease progression levels were significant for all estimated leaf traitsaccording to ANOVA. Mean disease severity (wilt) ratings are as follows: where 0 = fully asymptomatic, 1 = 1 to 25% foliar crown wilt, 2 = 26 to 50%, 3 = 51 to 75%, 4 = 76 to 99% and 5 = plants being completely dead (100% wilted).

All nine modeled leaf chemistries had significant differences between Ceratocystis wilt disease progression levels according to an ANOVA (Fig 4). Tannins, chlorophyll a+b, NSC, total C, leaf water, and phenols all decreased as ROD symptoms progressed (Fig 4). Cellulose was the only trait to increase with advancing disease severity levels. Trait variability within each disease severity level increased positively, except for chlorophyll a+b and lignin (Fig 4). This variability often peaked at disease severity level 2 and 3, with traits converging as the clones continued to wilt and senesce.

**Fig 5:**
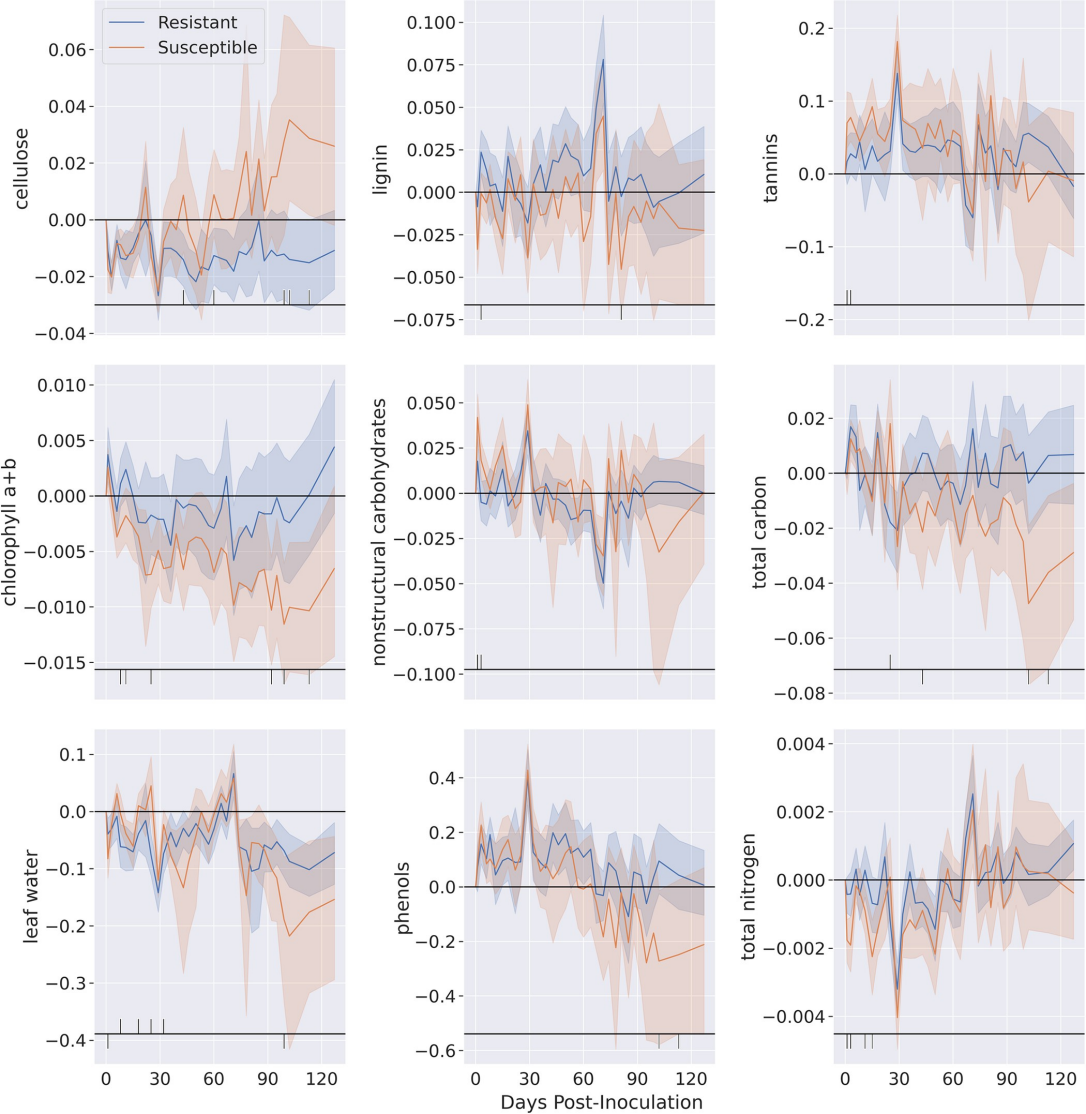
*Metrosideros polymorpha* leaf chemistry changes over time after artificial inoculation with *Ceratocystis lukuohia*. Chemistry changes are shown as deviation from values estimate from pre-inoculation leaf spectra. Blue lines represent *Resistant* individuals (disease severity rating of 0 throughout the trial duration), while orange lines represent *Susceptible* individuals (those with disease severity ratings from 1 to 4) at a time when disease progression levels where 0 and 1. All data were normalized using control plants. Leaf chemical values with significant differences according to a t-test are depicted by black lines at the bottom of each graph. Lines above the horizontal black line represent times when resistant plants were greater in a particular trait than susceptible plants, and ticks below represent the reverse.

To further investigate how Ceratocystis wilt symptoms appear in early disease progression stages, we compared leaf traits of *Susceptible* clones in the early stages of symptom expression (disease severity levels 0 and 1) with *Resistant* saplings (Fig 5). Patterns for cellulose showed decreases in both groups post-inoculation. While *Susceptible* individuals showed increased cellulose above pre-inoculation levels, *Resistant* individuals showed persistently low cellulose (Fig 5). Spectral patterns for lignin were consistent across trials for both groups but were lower in *Susceptible* individuals at only two sampling points (Fig 5). Both tannins and phenols increased post-inoculation in all plants, with levels returning to pre-inoculation levels by the end of the trial period (Fig 5). *Susceptible* individuals had lower phenol levels than both their pre-inoculation levels and *Resistant* plants at the end of the trial period. Chlorophyll a+b divergence between *Susceptible* and *Resistant* clones could be observed almost immediately after inoculation, with *Resistant* clones maintaining higher chlorophyll a+b levels throughout the sampling period (Fig 4). For both resistant and *Susceptible* individuals, NSCs remained near pre-inoculation levels, with some initial spikes in the first month of the inoculation trial (Fig 5). Total C decreased in *Susceptible* clones approximately one month following inoculations and continued declining throughout the sampling period (Fig 5). *Resistant* plants maintained relatively constant total C. Leaf water declined slightly post-inoculation in both groups, and divergence between pre- and post-inoculation levels increased around day 75 (Fig 5). Lastly, total N remained near pre-inoculation levels in both groups, with a notable negative spike around day 30 that corresponds to positive spikes in phenols, NSCs, and tannins (Fig 5).

## Discussion

In this study, we were able to: 1) identify key spectral differences between pre-inoculation *Susceptible* and *Resistant M*. *polymorpha* individuals; and 2) examine the post-inoculation progression of changing foliar chemistries across resistance conditions, varieties, and site variables. When we controlled for variation associated with *M*. *polymorpha* variety and site, we were able to use leaf-level spectroscopic measurements and modeled chemistries to detect disease resistance signals in reflectance data. By nondestructively sampling reflectance data and converting to chemical trait estimates, we were able to understand host response to *C*. *lukuohia* infection. We observed declines in six of the nine leaf traits following inoculation, and an increase in one trait (cellulose), as symptoms progressed in susceptible plants. Asner et al. found similar *in situ* changes in leaves collected from healthy canopies killed by *Ceratocystis* [50]. Further, by comparing the derived chemical response of *Resistant* and early-stage *Susceptible* plants, we gained insight into the mechanisms of resistance, discussed below, to *C*. *lukuohia*. Taken together, these analyses provide promising results for next stage plane based remote sensing studies of disease resistence in native *M*. *polymorpha* forests.

We demonstrated that resistance to Ceratocystis wilt of ʻōhiʻa is detectable in leaf-level reflectance data by applying a two-way ANOVA to PCs of reflectance measurements collected prior to inoculations. With the exception of the STBK site, we identified at least one PC that separated individuals based on their susceptibility to *C*. *lukuohia* for each taxonomic variety and site grouping. Site of origin and taxonomic classification as well as their interaction with resistance had significant effects on many of the PCs that separated the data based on resistance. As a result, it is important to consider the effect that genetic variation has on reflectance data when assessing resistance. This conclusion is confirmed by prior studies that have demonstrated the strong influence of both taxonomic classification and environmental filtering on leaf spectra of *M*. *polymorpha* grown in common gardens [70]. *M*. *polymorpha* is dominant across many ecological niches, and environmental filtering plays an important role in selecting for specific varieties of this genetically-diverse species [64, 77, 78]. This, in turn, has resulted in heritable differences in reflectance spectra of *M*. *polymorpha* across environmental gradients [70]. Disease resistance screening using spectroscopy is in early stages of development, but this technique has been demonstrated to successfully distinguish between resistant and susceptible hosts in a number of forest and agricultural plant species [16, 20, 34, 36]. This study demonstrates that a resistance signal to *C*. *lukuohia* infection is present in leaf reflectance data, but because of the influence of site and genetic factors on leaf spectra, it is important to continue considering these factors in future investigations.

*M*. *polymorpha* leaf traits not only responded to *C*. *lukuohia* inoculations, but *Resistant* and early-stage *Susceptible* individuals differed in their leaf trait responses, indicating that response mechanisms differ between these individuals. Lignin and cellulose are structural molecules found in plant cell walls. As lignin levels in hosts have not been found to be responsive to *Ceratocystis* infection [21, 50], its lack of change across the disease severity levels was not surprising. In contrast, leaf cellulose increased in *Susceptible* individuals as disease progression levels advanced while leaf cellulose decreased in *Resistant* plants. In *Arabidopsis thaliana*, mutants with inhibited cellulose synthesis demonstrated greater resistance to fungal pathogens, likely due to upregulated production of antimicrobial secondary metabolites, demonstrating that inhibited cellulose synthesis may be indicative of novel defense pathways and increased disease resistance [79]. Due to the lower leaf cellulose levels in *Resistant M*. *polymorpha* individuals compared to early-stage *Susceptible* plants and the increase in cellulose across the disease progression levels, we hypothesize that a novel defense pathway may be present in resistant *M*. *polymorpha* but not susceptible individuals.

In response to plant stress caused by drought or pathogens, NSC production increases initially, but long-term stress depletes NSC levels as growth slows relative metabolic functions [80–82]. In symptomatic (i.e. wilted) canopies of mature *M*. *polymorpha* trees infected by *C*. *lukuohia*, NSC concentrations can decrease by 30–45% compared to asymptomatic canopies [50]. For both *Resistant* and *Susceptible* individuals, leaf NSC levels spiked three times in the first month, suggesting that both groups demonstrated a stress response to *C*. *lukohia* inoculation. *In situ*, green canopies in the early stages of responding to infections likewise had higher NSC concentrations [21]. As disease symptoms progressed in susceptible individuals, NSC concentrations decreased as metabolic functions demanded more energy than was being produced and stored as NSC.

Phenols and tannins are compounds that support a plant’s secondary defense mechanisms, the production of which can be induced by infection [83, 84]. In some species, high phenol levels are indicative of disease resistance [85, 86]. We observed a disease-induced response in both tannins and phenols for all plants. *In situ* estimates of secondary compounds in wilted canopies of *M*. *polymorpha* likewise showed an increase in phenols, though there was no response in tannin levels [21]. In this study, following the initial response to inoculation, phenols and tannins decreased as the plants senesced.

Chlorophyll a+b declined in early-stage susceptible plants. This decline likely induced the decrease in total C observed in *Resistant* plants as disease-induced chlorophyll reductions can lead to impaired photosynthesis and therefore carbohydrate synthesis [87, 88]. Chlorophyll degradation in susceptible, diseased hosts has led to successful early detection of agricultural diseases using chlorophyll fluorescence [89, 90]. Chlorophyll fluorescence has additionally been used to screen for disease resistant hosts in agricultural crops as florescence characteristics differ between resistant and susceptible individuals [91–93]. Plant classified as *Resistant* in our study maintained consistent chlorophyll a+b and total C levels throughout the inoculation trial. Due to the differential response of chlorophyll a+b in *Resistant* and *Susceptible* plants, chlorophyll fluorescence may be a useful screening mechanism for the ʻŌDRP.

Overall, six of the nine chemistries we estimated decreased, and one, cellulose, increased across the six severity levels for rating *C*. *lukuohia-*induced symptom expression. At disease severity levels 2 and 3 (26–76% wilt), we found high variability in those seven chemistries, suggesting that leaf chemistry is rapidly changing at this point in symptom progression. At the canopy level, Weingarten et al. found that leaf water content, percent N, chlorophyll a+b, NSC, phenols, cellulose, and leaf water differed between healthy green *M*. *polymorpha* canopies and those that were asymptomatic and later became wilted [21]. While our study confirmed that these traits change prior to canopy browning, we found only short-term increases in NSC and phenols and that these traits decreased in later stages of plant decline and death. Unlike Weingarten et al. [21], which only assessed canopies in earlier stages of wilting, we also detected decreases in tannins and total C in response to *C*. *lukuohia* inoculation, though the decline in tannins was confirmed by Asner et al. when comparing healthy and senesced canopies *in situ* [50].

As with with other aggressive forest diseases, it is necessary to detect infected individuals, understand how the pathogen affects the host, and then identify resistant individuals. The potential of using remote sensing to screen for resistant individuals has been demonstrated in both agricultural and forest systems [16, 20, 34, 36]. Here, we demonstrate that resistance in *M*. *polymorpha* to *C*. *lukuohia* is detectable using VSWIR spectroscopy. Due to the sensitivity of reflectance data to genetic factors, these must be considered in resistance screening programs. By sampling *M*. *polymorpha* leaf reflectance throughout the inoculation trials, we demonstrated the ability of these data to non-destructively monitor changes in leaf traits over time. We observed changes in seven of the nine estimated traits throughout symptom progression. Further, we observed different responses in *Resistant* and early-stage *Susceptible* clones, most notably in chlorophyll a+b and cellulose. Future applications of spectroscopy to forest pathogens will allow us to address questions related to some of the most problematic diseases.

## Supporting information

S1 Fig Example reflectance spectra of a) *Resistant* and b) *Susceptible* individuals throughout the inoculation trials. Spectra collected prior to inoculations (Pre) are bolded black lines, and the last spectra collected (Post) are bolded blue. All reflectance data collected between the initial and final collection are represented as light grey lines.
(TIF)

S1 Table Soil substrate age and elevation at the four sites where mother trees are located. (DOCX)

S2 Table Results of a two-way ANOVA exploring the effect of site and variety on mother tree survival rate. (DOCX)

S3 Table ANOVA and pairwise Tukey results assessing separability of *M*. *polymorpha* varieties using principal components (PC) of leaf reflectance data. Only significant PCs according to the ANOVA are displayed. Variety pairs differentiable according to pairwise Tukey HSD are highlighted.
(DOCX)

S4 Table ANOVA and pairwise Tukey results assessing separability of greenhouse-grown individuals collected from different sites using principal components (PC) of leaf reflectance data. Only the first three significant PCs according to the ANOVA are displayed. Variety pairs differentiable according to pairwise Tukey are highlighted.
(DOCX)

## References

[1] Bright C. Invasive Species: Pathogens of Globalization. Foreign Policy. 1999;(116):50–64.

[2] Ramsfield TD, Bentz BJ, Faccoli M, Jactel H, Brockerhoff EG. Forest health in a changing world: effects of globalization and climate change on forest insect and pathogen impacts. Forestry. 2016 Jul;89(3):245–52.

[3] Simler-Williamson AB, Rizzo DM, Cobb RC. Interacting Effects of Global Change on Forest Pest and Pathogen Dynamics. Annu Rev Ecol Evol Syst. 2019 Nov 2;50(1):381–403.

[4] Dukes JS, Pontius J, Orwig D, Garnas JR, Rodgers VL, Brazee N, et al. Responses of insect pests, pathogens, and invasive plant species to climate change in the forests of northeastern North America: What can we predict? Canadian Journal of Forest Research. 2009 Feb;39(2):231–48.

[5] Grulke NE. The nexus of host and pathogen phenology: understanding the disease triangle with climate change. New Phytologist. 2011 Jan;189(1):8–11. doi: 10.1111/j.1469-8137.2010.03568.x 21166095

[6] Levine JM, D’Antonio CM. Forecasting Biological Invasions with Increasing International Trade. Conservation Biology. 2003;17(1):322–6.

[7] Burdon RD. Genetic diversity and disease resistance: some considerations for research, breeding, and employment. Canadian Journal of Forest Research. 2001 Apr;31(4):596.

[8] Schaberg PG, DeHayes DH, Hawley GJ, Nijensohn SE. Anthropogenic alterations of genetic diversity within tree populations: Implications for forest ecosystem resilience. Forest Ecology and Management. 2008 Aug;256(5):855–62.

[9] Freer-Smith PH, Webber JF. Tree pests and diseases: the threat to biodiversity and the delivery of ecosystem services. Biodivers Conserv. 2017 Dec;26(13):3167–81.

[10] Haight RG, Homans FR, Horie T, Mehta SV, Smith DJ, Venette RC. Assessing the Cost of an Invasive Forest Pathogen: A Case Study with Oak Wilt. Environmental Management. 2011 Mar;47(3):506–17. doi: 10.1007/s00267-011-9624-5 21331653

[11] Loope L, Hughes F, Keith L, Harrington R, Hauff R, Friday JB, et al. Guidance document for Rapid Ohia Death: background for the 2017–2019 ROD Strategic Response Plan. University of Hawaii: College of Tropical Agriculture and Human Resources. 2016;

[12] Sniezko RA. Resistance breeding against nonnative pathogens in forest trees—current successes in North America. Canadian Journal of Plant Pathology. 2006 Mar;28(sup1):S270–9.

[13] Townsend AM, Bentz SE, Douglass LW. Evaluation of 19 American Elm Clones for Tolerance to Dutch Elm Disease. Journal of Environmental Horticulture. 2005 Mar 1;23(1):21–4.

[14] Sniezko RA, Koch J. Breeding trees resistant to insects and diseases: putting theory into application. Biol Invasions. 2017 Nov 1;19(11):3377–400.

[15] Conrad AO, Bonello P. Application of Infrared and Raman Spectroscopy for the Identification of Disease Resistant Trees. Frontiers in Plant Science [Internet]. 2016 [cited 2022 Aug 5];6. Available from: https://www.frontiersin.org/articles/10.3389/fpls.2015.01152 26779211 10.3389/fpls.2015.01152 PMC4703757

[16] Martin JA, Solla A, Woodward S, Gil L. Fourier transform-infrared spectroscopy as a new method for evaluating host resistance in the Dutch elm disease complex. Tree Physiology. 2005 Oct 1;25(10):1331–8. doi: 10.1093/treephys/25.10.1331 16076781

[17] Martin JA, Solla A, Coimbra MA, Gil L. Metabolic fingerprinting allows discrimination between Ulmus pumila and U. minor, and between U. minor clones of different susceptibility to Dutch elm disease. Forest Pathology. 2008;38(4):244–56.

[18] Cotrozzi L. Spectroscopic detection of forest diseases: a review (1970–2020). J For Res. 2022 Feb 1;33(1):21–38.

[19] Perroy RL, Hughes M, Keith LM, Collier E, Sullivan T, Low G. Examining the Utility of Visible Near-Infrared and Optical Remote Sensing for the Early Detection of Rapid ‘Ōhi‘a Death. Remote Sensing. 2020 Jan;12(11):1846.

[20] Villari C, Dowkiw A, Enderle R, Ghasemkhani M, Kirisits T, Kjær ED, et al. Advanced spectroscopy-based phenotyping offers a potential solution to the ash dieback epidemic. Sci Rep. 2018 Dec;8(1):17448. doi: 10.1038/s41598-018-35770-0 30487524 PMC6262010

[21] Weingarten E, Martin R, Hughes F, Vaughn N, Schafron E, Asner GP. Early Detection of a Tree Pathogen using Airborne Remote Sensing. Ecological Applications. 2021;21(2). 10.1002/eap.2519 34918400

[22] Hansen MC, Potapov PV, Moore R, Hancher M, Turubanova SA, Tyukavina A, et al. High-Resolution Global Maps of 21st-Century Forest Cover Change. Science. 2013 Nov 15;342(6160):850–3. doi: 10.1126/science.1244693 24233722

[23] Turner W, Spector S, Gardiner N, Fladeland M, Sterling E, Steininger M. Remote sensing for biodiversity science and conservation. Trends in Ecology & Evolution. 2003 Jun 1;18(6):306–14.

[24] Balzotti CS, Asner GP, Adkins ED, Parsons EW. Spatial drivers of composition and connectivity across endangered tropical dry forests. Journal of Applied Ecology. 2020;57(8):1593–604.

[25] Torabzadeh H, Leiterer R, Hueni A, Schaepman ME, Morsdorf F. Tree species classification in a temperate mixed forest using a combination of imaging spectroscopy and airborne laser scanning. Agricultural and Forest Meteorology. 2019 Dec 15;279:107744.

[26] Trier ØD, Salberg AB, Kermit M, Rudjord Ø, Gobakken T, Næsset E, et al. Tree species classification in Norway from airborne hyperspectral and airborne laser scanning data. European Journal of Remote Sensing. 2018 Jan 1;51(1):336–51.

[27] Féret JB, Asner GP. Mapping tropical forest canopy diversity using high-fidelity imaging spectroscopy. Ecological Applications. 2014;24(6):1289–96. 29160652 10.1890/13-1824.1

[28] Blonder B, Graae BJ, Greer B, Haagsma M, Helsen K, Kapás RE, et al. Remote sensing of ploidy level in quaking aspen (Populus tremuloides Michx.). Journal of Ecology. 2020;108(1):175–88.

[29] Cavender-Bares J, Meireles JE, Couture JJ, Kaproth MA, Kingdon CC, Singh A, et al. Associations of Leaf Spectra with Genetic and Phylogenetic Variation in Oaks: Prospects for Remote Detection of Biodiversity. Remote Sensing. 2016 Mar;8(3):221.

[30] Asner GP, Martin RE. Airborne spectranomics: mapping canopy chemical and taxonomic diversity in tropical forests. Frontiers in Ecology and the Environment. 2009;7(5):269–76.

[31] Asner GP, Martin RE. Spectranomics: Emerging science and conservation opportunities at the interface of biodiversity and remote sensing. Global Ecology and Conservation. 2016 Oct 1;8:212–9.

[32] Palmer MW, Wohlgemuth T, Earls P, Arevalo JR, Thompson S. Opportunities for long-term ecological research at the Tallgrass Prairie Preserve, Oklahoma. Proceedings of the ILTER Regional Workshop: Cooperation in Long Term Ecological Research in Central and Eastern Europe, Budapest, Hungary. 2000;22.

[33] Rocchini D, Santos MJ, Ustin SL, Féret JB, Asner GP, Beierkuhnlein C, et al. The Spectral Species Concept in Living Color. Journal of Geophysical Research: Biogeosciences [Internet]. 2022 [cited 2022 Sep 7];127(9). Available from: http://onlinelibrary.wiley.com/doi/abs/10.1029/2022JG007026 36247363 10.1029/2022JG007026 PMC9539608

[34] Munaiz ED, Townsend PA, Havey MJ. Reflectance Spectroscopy for Non-Destructive Measurement and Genetic Analysis of Amounts and Types of Epicuticular Waxes on Onion Leaves. Molecules. 2020 Jan;25(15):3454. doi: 10.3390/molecules25153454 32751296 PMC7436246

[35] Pandey P, Payn K, Lu Y, Heine A, Walker T, Young S. High Throughput Phenotyping for Fusiform Rust Disease Resistance in Loblolly Pine Using Hyperspectral Imaging. In: American Society of Agricultural and Biological Engineers [Internet]. 2020 [cited 2022 Aug 10]. Available from: 10.13031/aim.202000872

[36] Sabatier D, Moon C, Mhora T, Rutherford R, Laing M. Near-infrared reflectance (NIR) spectroscopy as a high-throughput screening tool for pest and disease resistance in a sugarcane breeding programme. International Sugar Journal. 2014 Jan 1;Volume 116:580–3.

[37] Su WH, Yang C, Dong Y, Johnson R, Page R, Szinyei T, et al. Hyperspectral imaging and improved feature variable selection for automated determination of deoxynivalenol in various genetic lines of barley kernels for resistance screening. Food Chemistry. 2021 May 1;343:128507. doi: 10.1016/j.foodchem.2020.128507 33160773

[38] Barnes I, Fourie A, Wingfield MJ, Harrington TC, McNew DL, Sugiyama LS, et al. New Ceratocystis species associated with rapid death of Metrosideros polymorpha in Hawai`i. 2002;40:28. 10.3767/persoonia.2018.40.07 PMC6146641 30505000

[39] Hughes F, Juzwik J, Harrington T, Keith L. Pathogenicity, Symptom Development, and Colonization of Metrosideros polymorpha by Ceratocystis lukuohia. Plant Disease. 2020;104(8):2233–41. doi: 10.1094/PDIS-09-19-1905-RE 32552282

[40] Mortenson LA, Hughes RF, Friday JB, Keith LM, Barbosa JM, Friday NJ, et al. Assessing spatial distribution, stand impacts and rate of Ceratocystis fimbriata induced ‘ōhi‘a (Metrosideros polymorpha) mortality in a tropical wet forest, Hawai‘i Island, USA. Forest Ecology and Management 377: 83–92. 2016;377:83–92.

[41] Pratt T, Atkinson C, Banko PC, Jacobi J, Woodworth B. Conservation Biology of Hawaiian Forest Birds [Internet]. Yale University Press; 2009 [cited 2022 Aug 4]. Available from: https://yalebooks.yale.edu/9780300141085/conservation-biology-of-hawaiian-forest-birds

[42] Kagawa A, Sack L, Duarte K, James S. Hawaiian native forest conserves water relative to timber plantation: species and stand traits influence water use. Ecol Appl. 2009 Sep;19(6):1429–43. doi: 10.1890/08-1704.1 19769092

[43] Chow ET. The Sovereign Nation of Hawai’i: Resistance in the Legacy of “Aloha ’Oe.” SUURJ: Seattle University Undergraduate Research Journal. 2018;2(15):15.

[44] Westervelt WD. Hawaiian Legends of Volcanoes (mythology). Ellis Press; 1916. 284 p.

[45] Brill E, Hughes MA, Heller WP, Keith LM. First Report of Ceratocystis lukuohia on Metrosideros polymorpha on the Island of Kaua‘i, Hawai‘i. Plant Disease. 2019 Nov;103(11):2961.

[46] Heller WP, Hughes MA, Luiz BC, Brill E, Friday JB, Williams AM, et al. First report of Ceratocystis huliohia causing mortality of Metrosideros polymorpha trees on the Island of Kauaʻi, Hawaiʻi USA. Forest Pathology. 2019;49(5):e12546.

[47] Luiz BC, Giardina CP, Keith LM, Jacobs DF, Sniezko RA, Hughes MA, et al. A framework for establishing a rapid ‘Ōhi‘a death resistance program. New Forests [Internet]. 2022 Jan 25 [cited 2022 Aug 10]; Available from: https://link.springer.com/10.1007/s11056-021-09896-5

[48] Camp RJ, LaPointe DA, Hart PJ, Sedgwick DE, Canale LK. Large-scale tree mortality from Rapid Ohia Death negatively influences avifauna in lower Puna, Hawaii Island, USA. The Condor. 2019 May 1;121(2):duz007.

[49] Fortini LB, Kaiser LR, Keith LM, Price J, Hughes RF, Jacobi JD, et al. The evolving threat of Rapid ‘Ōhi‘a Death (ROD) to Hawai‘i’s native ecosystems and rare plant species. Forest Ecology and Management. 2019 Sep;448:376–85.

[50] Asner GP, Martin RE, Keith LM, Heller WP, Hughes MA, Vaughn NR, et al. A Spectral Mapping Signature for the Rapid Ohia Death (ROD) Pathogen in Hawaiian Forests. Remote Sensing. 2018 Mar;10(3):404.

[51] Hughes M, Luiz B, Keith L, Giardina C. The Development of a Resistance Screening Program of Ohia to Ceratocystis Pathogens Causing Widespread Mortality on Hawaii Island. 2020;19–19.

[52] Roy K, Jaenecke KA, Peck RW. Ambrosia Beetle (Coleoptera: Curculionidae) Communities and Frass Production in ‘Ōhi’a (Myrtales: Myrtaceae) Infected With Ceratocystis (Microascales: Ceratocystidaceae) Fungi Responsible for Rapid ‘Ōhi’a Death. Environ Entomol. 2020 Dec 14;49(6):1345–54. 33315073 10.1093/ee/nvaa108

[53] D’Antonio CM, Vitousek PM. Biological Invasions by Exotic Grasses, the Grass/Fire Cycle, and Global Change. Annual Review of Ecology and Systematics. 1992;23(1):63–87.

[54] Somers B, Asner GP. Hyperspectral Time Series Analysis of Native and Invasive Species in Hawaiian Rainforests. Remote Sensing. 2012 Sep;4(9):2510–29.

[55] Vitousek PM, Walker LR, Whiteaker LD, Mueller-Dombois D, Matson PA. Biological invasion by *Myrica faya* alters ecosystem development in Hawaii. Science. 1987;238(4828):802–804. 17814707 10.1126/science.238.4828.802

[56] Povak NA, Hessburg PF, Giardina CP, Reynolds KM, Heider C, Salminen E, et al. A watershed decision support tool for managing invasive species on Hawai‘i Island, USA. Forest Ecology and Management. 2017 Sep;400:300–20.

[57] Boehmer HJ, Wagner HH, Jacobi JD, Gerrish GC, Mueller-Dombois D. Rebuilding after collapse: evidence for long-term cohort dynamics in the native Hawaiian rain forest. Journal of Vegetation Science. 2013;24(4):639–50.

[58] Jacobi JD, Gerrish G, Mueller-Dombois D. ‘Ohi’a Dieback in Hawai’i: Vegetation Changes in Permanent Plots. Pacific Science. 1983;37(4).

[59] Strauch AM, Giardina CP, MacKenzie RA, Heider C, Giambelluca TW, Salminen E, et al. Modeled Effects of Climate Change and Plant Invasion on Watershed Function Across a Steep Tropical Rainfall Gradient. Ecosystems. 2017 Apr 1;20(3):583–600.

[60] Perroy RL, Sullivan T, Benitez D, Hughes RF, Keith LM, Brill E, et al. Spatial Patterns of ‘Ōhi‘a Mortality Associated with Rapid ‘Ōhi‘a Death and Ungulate Presence. Forests. 2021 Aug 4;12(8):1035.

[61] Vaughn NR, Asner GP, Brodrick PG, Martin RE, Heckler JW, Knapp DE, et al. An Approach for High-Resolution Mapping of Hawaiian Metrosideros Forest Mortality Using Laser-Guided Imaging Spectroscopy. Remote Sensing. 2018 Apr;10(4):502.

[62] Choi JY, Purugganan M, Stacy EA. Divergent Selection and Primary Gene Flow Shape Incipient Speciation of a Riparian Tree on Hawaii Island. Molecular Biology and Evolution. 2020 Mar 1;37(3):695–710. doi: 10.1093/molbev/msz259 31693149 PMC7038655

[63] Dawson J, Stemmermann L. Metrosideros (Gaud). In: Manual of the Flowering Plants of Hawai’i. Honoluu, HI: Univ. Hawai’i Press; 1990. p. 964–70.

[64] Stacy EA, Johansen JB, Sakishima T, Price DK, Pillon Y. Incipient radiation within the dominant Hawaiian tree Metrosideros polymorpha. Heredity. 2014 Oct;113(4):334–42. doi: 10.1038/hdy.2014.47 24824285 PMC4181069

[65] Stacy EA, Johansen JB, Sakishima T, Price DK. Genetic analysis of an ephemeral intraspecific hybrid zone in the hypervariable tree, Metrosideros polymorpha, on Hawai‘i Island. Heredity. 2016 Sep;117(3):173–83. doi: 10.1038/hdy.2016.40 27301333 PMC4981685

[66] Kitayama K. Ecological and Genetic Implications of Foliar Polymorphism inMetrosideros polymorphaGaud. (Myrtaceae) in a Habitat Matrix on Mauna Loa, Hawaii. Annals of Botany. 1997 Oct;80(4):491–7.

[67] James SA, Puttock CF, Cordell S, Adams RP. Morphological and genetic variation within Metrosideros polymorpha (Myrtaceae) on Hawai’i. New Zealand Journal of Botany. 2004 Jun 1;42(2):263–70.

[68] Keith LM, Hughes RF, Sugiyama LS, Heller WP, Bushe BC, Friday JB. First Report of Ceratocystis Wilt on ˋŌhiˋa (Metrosideros polymorpha). Plant Disease. 2015 Sep;99(9):1276.

[69] Hueni A, Bialek A. Cause, Effect, and Correction of Field Spectroradiometer Interchannel Radiometric Steps. IEEE Journal of Selected Topics in Applied Earth Observations and Remote Sensing. 2017 Apr;10(4):1542–51.

[70] Martin RE, Asner GP, Sack L. Genetic variation in leaf pigment, optical and photosynthetic function among diverse phenotypes of Metrosideros polymorpha grown in a common garden. Oecologia. 2007 Mar 1;151(3):387–400. doi: 10.1007/s00442-006-0604-z 17124568

[71] Feret JB, Asner GP. Tree Species Discrimination in Tropical Forests Using Airborne Imaging Spectroscopy. IEEE Transactions on Geoscience and Remote Sensing. 2013 Jan;51(1):73–84.

[72] Virtanen P, Gommers R, Oliphant TE, Haberland M, Reddy T, Cournapeau D, et al. SciPy 1.0: fundamental algorithms for scientific computing in Python. Nat Methods. 2020 Mar 2;17(3):261–72. doi: 10.1038/s41592-019-0686-2 32015543 PMC7056644

[73] Draper FC, Baraloto C, Brodrick PG, Phillips OL, Martinez RV, Coronado ENH, et al. Imaging spectroscopy predicts variable distance decay across contrasting Amazonian tree communities. Journal of Ecology. 2019;107(2):696–710.

[74] Huesca M, García M, Roth KL, Casas A, Ustin SL. Canopy structural attributes derived from AVIRIS imaging spectroscopy data in a mixed broadleaf/conifer forest. Remote Sensing of Environment. 2016 Sep 1;182:208–26.

[75] Seabold S, Perktold J. statsmodels: Econometric and statistical modeling with python. In 2010.

[76] Asner GP, Martin RE, Anderson CB, Knapp DE. Quantifying forest canopy traits: Imaging spectroscopy versus field survey. Remote Sensing of Environment. 2015 Mar 1;158:15–27.

[77] Cordell S, Goldstein G, Mueller-Dombois D, Webb D, Vitousek PM. Physiological and morphological variation in Metrosideros polymorpha, a dominant Hawaiian tree species, along an altitudinal gradient: the role of phenotypic plasticity. Oecologia. 1998 Jan 1;113(2):188–96. doi: 10.1007/s004420050367 28308196

[78] Joel G, Aplet G, Vitousek PM. Leaf Morphology Along Environmental Gradients in Hawaiian Metrosideros Polymorpha. Biotropica. 1994;26(1):17–22.

[79] Hernández-Blanco C, Feng DX, Hu J, Sánchez-Vallet A, Deslandes L, Llorente F, et al. Impairment of Cellulose Synthases Required for Arabidopsis Secondary Cell Wall Formation Enhances Disease Resistance. The Plant Cell. 2007 Mar 1;19(3):890–903. doi: 10.1105/tpc.106.048058 17351116 PMC1867366

[80] Hartmann H, Trumbore S. Understanding the roles of nonstructural carbohydrates in forest trees–from what we can measure to what we want to know. New Phytologist. 2016;211(2):386–403. doi: 10.1111/nph.13955 27061438

[81] Klein T, Hoch G, Yakir D, Körner C. Drought stress, growth and nonstructural carbohydrate dynamics of pine trees in a semi-arid forest. Tree Physiology. 2014 Sep 1;34(9):981–92. doi: 10.1093/treephys/tpu071 25187568

[82] Tomasella M, Petrussa E, Petruzzellis F, Nardini A, Casolo V. The Possible Role of Non-Structural Carbohydrates in the Regulation of Tree Hydraulics. International Journal of Molecular Sciences. 2020 Jan;21(1):144. 10.3390/ijms21010144 PMC6981889 31878253

[83] Witzell J, Martín JA. Phenolic metabolites in the resistance of northern forest trees to pathogens —past experiences and future prospects. Can J For Res. 2008 Nov;38(11):2711–27.

[84] Zúñiga E, Luque J, Martos S. Lignin biosynthesis as a key mechanism to repress Polystigma amygdalinum, the causal agent of the red leaf blotch disease in almond. Journal of Plant Physiology. 2019 May;236:96–104. doi: 10.1016/j.jplph.2019.03.004 30939334

[85] Ewané CA, Lepoivre P, de Bellaire L de L, Lassois L. Involvement of phenolic compounds in the susceptibility of bananas to crown rot. A review. Biotechnol Agron Soc Environ. 2012;16(3).

[86] Reichel T, de Resende MLV, Monteiro ACA, Freitas NC, dos Santos Botelho DM. Constitutive Defense Strategy of Coffee Under Field Conditions: A Comparative Assessment of Resistant and Susceptible Cultivars to Rust. Mol Biotechnol. 2022 Mar 1;64(3):263–77. doi: 10.1007/s12033-021-00405-9 34595725

[87] Pinto LSRC, Azevedo JL, Pereira JO, Vieira MLC, Labate CA. Symptomless infection of banana and maize by endophytic fungi impairs photosynthetic efficiency. The New Phytologist. 2000 Sep;147(3):609–15. doi: 10.1046/j.1469-8137.2000.00722.x 33862932

[88] Yahya M, Saeed NA, Nadeem S, Hamed M, Saleem K. Effect of leaf rust disease on photosynthetic rate, chlorophyll contents and grain yield of wheat. Archives of Phytopathology and Plant Protection. 2020 May 27;53(9–10):425–39.

[89] Atta BM, Saleem M, Ali H, Arshad HMI, Ahmed M. Chlorophyll as a biomarker for early disease diagnosis. Laser Phys. 2018 May 9;28(6):065607.

[90] Christen D, Schönmann S, Jermini M, Strasser RJ, Défago G. Characterization and early detection of grapevine (Vitis vinifera) stress responses to esca disease by in situ chlorophyll fluorescence and comparison with drought stress. Environmental and Experimental Botany. 2007 Jul;60(3):504–14.

[91] Chaerle L, Hagenbeek D, De Bruyne E, Valcke R, Van Der Straeten D. Thermal and Chlorophyll-Fluorescence Imaging Distinguish Plant-Pathogen Interactions at an Early Stage. Plant and Cell Physiology. 2004 Jul 15;45(7):887–96. doi: 10.1093/pcp/pch097 15295072

[92] Durães FOM, Gama E, Magalhães P, Marriel I, Casela C, Oliveira A, et al. The usefulness of chlorophyll fluorescence in screening for disease resistance, water stress tolerance, aluminum toxicity, and use efficiency in maize. 2001 Jan 1; 356–360.

[93] Suárez JC, Vanegas JI, Contreras AT, Anzola JA, Urban MO, Beebe SE, et al. Chlorophyll Fluorescence Imaging as a Tool for Evaluating Disease Resistance of Common Bean Lines in the Western Amazon Region of Colombia. Plants. 2022 Jan;11(10):1371. doi: 10.3390/plants11101371 35631796 PMC9143997

