# Supplementary figures and images for: Leaf spectroscopy of resistance to Ceratocystis wilt of ‘Ōhi’a
Source: PLoS One. 2023 Jun 23;18(6):e0287144. doi: 10.1371/journal.pone.0287144 (PMC10289452; doi:10.1371/journal.pone.0287144)

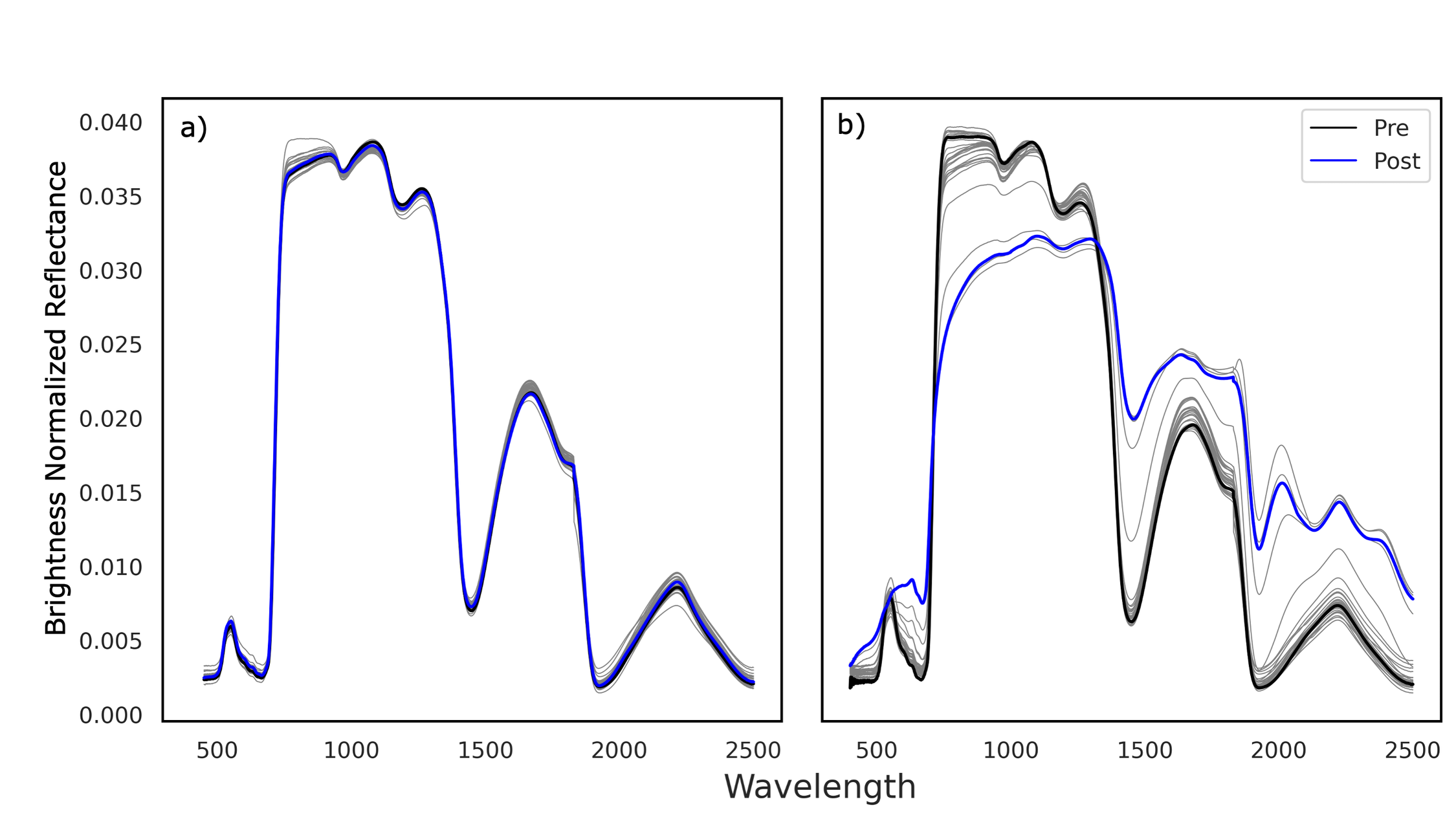

Supplement: S1 Fig — Example reflectance spectra of a) Resistant and b) Susceptible individuals throughout the inoculation trials. Spectra collected prior to inoculations (Pre) are bolded black lines, and the last spectra collected (Post) are bolded blue. All reflectance data collected between the initial and final collection are represented as light grey lines. (TIF) [file pone.0287144.s001.tif]
