# Supplementary material for: Leaf spectroscopy of resistance to Ceratocystis wilt of ‘Ōhi’a
Source: PLoS One. 2023 Jun 23;18(6):e0287144. doi: 10.1371/journal.pone.0287144 (PMC10289452; doi:10.1371/journal.pone.0287144)
Supplement: S1 Table — (DOCX) [file pone.0287144.s002.docx]

**S1 Table. Soil substrate age and elevation at the four sites where mother trees are located.**

| **Site** | **Soil substrate age (millions of years)** | **Elevation (meters)** |
| --- | --- | --- |
| KEMR | 750-1500 | 20 |
| IPIF | 750-1500 | 110 |
| STBK | 200-750 | 400 |
| PUKA | 400-750 | 280 |
