# Supplementary material for: Leaf spectroscopy of resistance to Ceratocystis wilt of ‘Ōhi’a
Source: PLoS One. 2023 Jun 23;18(6):e0287144. doi: 10.1371/journal.pone.0287144 (PMC10289452; doi:10.1371/journal.pone.0287144)
Supplement: S2 Table — (DOCX) [file pone.0287144.s003.docx]

**S2 Table. Results of a two-way ANOVA exploring the effect of site and variety on mother tree survival rate.**

| **Factor** | **p-value** |
| --- | --- |
| Site | 0.059 |
| Variety | 0.147 |
| Site x Variety | 0.110 |
