# Supplementary material for: Leaf spectroscopy of resistance to Ceratocystis wilt of ‘Ōhi’a
Source: PLoS One. 2023 Jun 23;18(6):e0287144. doi: 10.1371/journal.pone.0287144 (PMC10289452; doi:10.1371/journal.pone.0287144)
Supplement: S3 Table — Only significant PCs according to the ANOVA are displayed. Variety pairs differentiable according to pairwise Tukey HSD are highlighted. (DOCX) [file pone.0287144.s004.docx]

**S3 Table. ANOVA and pairwise Tukey results assessing separability of *M. polymorpha* varieties using principal components (PC) of leaf reflectance data.** Only significant PCs according to the ANOVA are displayed. Variety pairs differentiable according to pairwise Tukey HSD are highlighted.

| ANOVA p-value | Variety 1 | Variety 2 | Mean Difference | P-adj | Lower | Upper | Reject H0 |
| --- | --- | --- | --- | --- | --- | --- | --- |
| Principal Component 2 | | | | | | | |
| p-value = 0.045 | glaberrima | hybrid | 0.002 | 0.769 | -0.005 | 0.009 | FALSE |
|  | glaberrima | incana | -0.007 | 0.088 | -0.015 | 0.001 | FALSE |
|  | hybrid | incana | -0.009 | 0.049 | -0.018 | 0.000 | TRUE |
| Principal Component 3 | | | | | | | |
| p-value = 0.018 | glaberrima | hybrid | 0.002 | 0.362 | -0.002 | 0.006 | FALSE |
|  | glaberrima | incana | 0.005 | 0.015 | 0.001 | 0.008 | TRUE |
|  | hybrid | incana | 0.003 | 0.397 | -0.002 | 0.007 | FALSE |
| Principal Component 5 | | | | | | | |
| p-value = 0.008 | glaberrima | hybrid | 0.002 | 0.008 | 0.001 | 0.004 | TRUE |
|  | glaberrima | incana | 0.001 | 0.208 | -0.001 | 0.003 | FALSE |
|  | hybrid | incana | -0.001 | 0.552 | -0.003 | 0.001 | FALSE |
