# Supplementary material for: Leaf spectroscopy of resistance to Ceratocystis wilt of ‘Ōhi’a
Source: PLoS One. 2023 Jun 23;18(6):e0287144. doi: 10.1371/journal.pone.0287144 (PMC10289452; doi:10.1371/journal.pone.0287144)
Supplement: S4 Table — Only the first three significant PCs according to the ANOVA are displayed. Variety pairs differentiable according to pairwise Tukey are highlighted. (DOCX) [file pone.0287144.s005.docx]

**S4 Table. ANOVA and pairwise Tukey results assessing separability of greenhouse-grown individuals collected from different sites using principal components (PC) of leaf reflectance data.** Only the first three significant PCs according to the ANOVA are displayed. Variety pairs differentiable according to pairwise Tukey are highlighted.

| ANOVA p-value | Site 1 | Site 2 | Mean Difference | P-adj | Lower | Upper | Reject H0 |
| --- | --- | --- | --- | --- | --- | --- | --- |
| Principal Component 1 | | | | | | | |
| p-value = 1.6e^-22^ | IPIF | KEMR | -0.063 | 0.001 | -0.081 | -0.045 | TRUE |
|  | IPIF | PUKA | -0.048 | 0.001 | -0.061 | -0.036 | TRUE |
|  | IPIF | STBK | -0.059 | 0.001 | -0.075 | -0.043 | TRUE |
|  | KEMR | PUKA | 0.014 | 0.131 | -0.003 | 0.031 | FALSE |
|  | KEMR | STBK | 0.004 | 0.900 | -0.016 | 0.023 | FALSE |
|  | PUKA | STBK | -0.011 | 0.236 | -0.025 | 0.004 | FALSE |
| Principal Component 3 | | | | | | | |
| p-value = 7.7e^-3^ | IPIF | KEMR | 0.000 | 0.900 | -0.004 | 0.005 | FALSE |
|  | IPIF | PUKA | 0.001 | 0.692 | -0.002 | 0.005 | FALSE |
|  | IPIF | STBK | -0.004 | 0.087 | -0.008 | 0.000 | FALSE |
|  | KEMR | PUKA | 0.001 | 0.900 | -0.003 | 0.005 | FALSE |
|  | KEMR | STBK | -0.004 | 0.155 | -0.009 | 0.001 | FALSE |
|  | PUKA | STBK | -0.005 | 0.003 | -0.009 | -0.001 | TRUE |
| Principal Component 4 | | | | | | | |
| p-value = 5.2e^-7^ | IPIF | KEMR | 0.0046 | 0.001 | 0.002 | 0.0071 | TRUE |
|  | IPIF | PUKA | 0.005 | 0.001 | 0.002 | 0.007 | FALSE |
|  | IPIF | STBK | -0.001 | 0.532 | -0.003 | 0.001 | FALSE |
|  | KEMR | PUKA | 0.000 | 0.900 | -0.002 | 0.002 | TRUE |
|  | KEMR | STBK | -0.006 | 0.001 | -0.008 | -0.003 | TRUE |
|  | PUKA | STBK | -0.005 | 0.001 | -0.008 | -0.002 | FALSE |
